# Low bicarbonate replacement fluid normalizes metabolic alkalosis during continuous veno-venous hemofiltration with regional citrate anticoagulation

**DOI:** 10.1186/s13613-021-00850-4

**Published:** 2021-04-23

**Authors:** Paul Köglberger, Sebastian J. Klein, Georg Franz Lehner, Romuald Bellmann, Andreas Peer, Daniel Schwärzler, Michael Joannidis

**Affiliations:** Division of Intensive Care and Emergency Medicine, Department of Internal Medicine, Medical University Innsbruck, Anichstr. 35, 6020 Innsbruck, Austria

**Keywords:** Metabolic alkalosis, Phoxilium^®^, Biphozyl^®^, Continuous veno-venous hemofiltration, Regional citrate anticoagulation, Acute kidney injury

## Abstract

**Background:**

Metabolic alkalosis is a frequently occurring problem during continuous veno-venous hemofiltration (CVVH) with regional citrate anticoagulation (RCA). This study aimed to evaluate the effectiveness of switching from high to low bicarbonate (HCO_3_^−^) replacement fluid in alkalotic critically ill patients with acute kidney injury treated by CVVH and RCA.

**Methods:**

A retrospective-comparative study design was applied. Patients who underwent CVVH with RCA in the ICU between 09/2016 and 11/2017 were evaluated. Data were available from the clinical routine. A switch of the replacement fluid Phoxilium^®^ (30 mmol/l HCO_3_^−^) to Biphozyl^®^ (22 mmol/l HCO_3_^−^) was performed as blood HCO_3_^−^ concentration persisted ≥ 26 mmol/l despite adjustments of citrate dose and blood flow. Data were collected from 72 h before the switch of the replacement solutions until 72 h afterwards.

**Results:**

Of 153 patients treated with CVVH during that period, 45 patients were switched from Phoxilium^®^ to Biphozyl^®^. Forty-two patients (42 circuits) were available for statistical analysis. After switching the replacement fluid from Phoxilium^®^ to Biphozyl^®^ the serum HCO_3_^−^ concentration decreased significantly from 27.7 mmol/l (IQR 26.9–28.9) to 25.8 mmol/l (IQR 24.6–27.7) within 24 h (*p* < *0.001*). Base excess (BE) decreased significantly from 4.0 mmol/l (IQR 3.1–5.1) to 1.8 mmol/l (IQR 0.2–3.4) within 24 h (*p* < *0.001).* HCO_3_^−^ and BE concentration remained stable from 24 h till the end of observation at 72 h after the replacement fluid change (*p* = *0.225*). pH and PaCO_2_ did not change significantly after the switch of the replacement fluid until 72 h.

**Conclusions:**

This retrospective analysis suggests that for patients developing refractory metabolic alkalosis during CVVH with RCA the use of Biphozyl^®^ reduces external HCO_3_^−^ load and sustainably corrects intracorporeal HCO_3_^−^ and BE concentrations. Future studies have to prove whether correcting metabolic alkalosis during CVVH with RCA in critically ill patients is of relevance in terms of clinical outcome.

**Supplementary Information:**

The online version contains supplementary material available at 10.1186/s13613-021-00850-4.

## Background

Acute kidney injury (AKI) frequently occurs in critically ill patients and is associated with both high morbidity and mortality [1–4]. The international AKI-EPI study found that up to 57% of ICU patients develop AKI, of whom approximately 25% require renal replacement therapy (RRT) [2]. When providing continuous renal replacement therapy (CRRT) anticoagulation is recommended to prevent blood clotting and extend the filter lifespan [5–7]. Regional citrate anticoagulation (RCA) is considered the method of choice preferred over the use of heparin in patients who have no contraindications against citrate [5, 8, 9]. Most replacement fluids available for continuous veno-venous (CVVH) were developed for use with heparin anticoagulation and therefore contain relatively high bicarbonate (HCO_3_^−^) levels. When applying RCA, additional HCO_3_^−^ is originating from citrate metabolism, often leading to metabolic alkalosis [10, 11], especially in combination with high bicarbonate-containing replacement fluids. High-bicarbonate solutions might even have a negative effect on mid-term mortality in AKI patients undergoing CVVH with RCA [12]. Phoxilium^®^ is used as a registered replacement fluid for CVVH in Europe and contains 30 mmol/l of HCO_3_^−^. Biphozyl^®^ was developed recently and registered as a replacement fluid for CVVH, containing only 22 mmol/l HCO_3_^−^. Therefore, Biphozyl^®^ should reduce bicarbonate load during CVVH using RCA. No studies have been published analysing Biphozyl^®^ yet.

Since the availability of Biphozyl^®^, we started to switch patients who developed persistent metabolic alkalosis during CVVH with RCA from Phoxilium^®^ to Biphozyl^®^. As most of the intracorporeal bicarbonate results from metabolized citrate during RCA, it was unclear, whether a reduction of exogenous bicarbonate delivered by the replacement fluid would normalize metabolic alkalosis in this setting.

Therefore, this retrospective cohort study of ICU patients with metabolic alkalosis during CVVH with RCA was aimed at demonstrating a sustained reduction of serum bicarbonate levels by switching to a low bicarbonate-containing replacement fluid without changing substitution rate or blood flow.

## Methods

### Study design and participants

This trial was of a retrospective-comparative study design, undertaken at the Medical Intensive Care Unit, Medical University of Innsbruck. Patients who previously underwent CVVH with RCA in the ICU between 09/2016 and 11/2017 were evaluated.

Patients were found eligible for inclusion, if they were (1)  ≥ 18 years, (2) admitted to Intensive Care Unit (ICU), had (3) an indication for CVVH as determined by the attending physician and (4) a change of replacement fluid from Phoxilium^®^ to Biphozyl^®^ because of persistent metabolic alkalosis defined by a blood HCO_3_^−^ concentration ≥ 26 mmol/l which could not be corrected by a reduction of citrate rate allowing a max. post-filter calcium of 0,45 mmol/l and a decrease of blood flow as long as filtration fraction remained below 30% while aiming at a ultrafiltration dose of 25 ml/kg/h. Patients were excluded in case of (1) CVVH duration less than 48 h and (2) < 12 h of CVVH treatment duration with Phoxilium^®^ or Biphozyl^®^.

### Outcomes

The primary objective of the study was to describe the reduction of elevated bicarbonate levels in metabolic alkalosis after switching from a high (30 mmol/l of HCO_3_^−^) to low (22 mmol/l HCO_3_^−^) bicarbonate-containing replacement fluid early after the switch (0 h to 24 h). The secondary objective of the study was to describe the influence of reduced bicarbonate administration on acid–base status till 72 h after the switch.

The primary outcome was the change in HCO_3_^−^ and BE levels 24 h after switching from Phoxilium^®^ to Biphozyl^®^.

The secondary outcomes were changes of serum HCO_3_^−^, BE, CO_2_ and pH values as well as differences of the pH between mechanical and non-mechanical ventilated patients between the switch of the replacement fluid (0 h) and 72 h thereafter. Furthermore, correlations were established between the acid–base parameters HCO_3_^−^, BE, CO_2_, pH, and the respiratory status (mechanical and non-mechanical ventilated) during the full study period (− 72 h to + 72 h). For safety reasons, changes of ionized serum calcium levels after the switch (between 0 and 72 h) were assessed.

### Patient management

The overall targeted observation period was from 72 h before the switch of the replacement solution until 72 h afterwards. All eligible patients received consecutively Phoxilium^®^ and Biphozyl^®^. The bicarbonate concentration is the main difference between the two replacement solutions. Unlike Phoxilium^®^, Biphozyl^®^ does not contain calcium. Slight differences are apparent in the concentration of the electrolytes magnesium, chloride and phosphate. A comparison of the two replacement solutions is provided in Table 1. Replacement fluid was administered post-filter at a targeted rate to achieve a dose of 25 ml/kg/h (including pre-filter citrate solution). Regional citrate anticoagulation was pre-filter infusion of Regiocit^®^ (Prismocitrate 18/0, Gambro Lundia AB, Sweden). For calcium replacement, a custom-made Ca^2+^ solution (calcium chloride 500 mmol/l, with or without magnesium chloride 250 mmol/l) was infused by a separate central venous line post-filter. Changes of the hemofiltration circuit (tubes, filter, etc.) were performed as necessary and according to the clinical routine, CVVH was conducted using Gambro PrismafleX^®^ eXeed Systems according to the manufacturers protocol and local standard operating procedures.

**Table 1:** Comparison of the composition of the reconstituted solutions

Active substances		Phoxilium^**®**^ (mmol/l)	Biphozyl^**®**^ (mmol/l)
Calcium	Ca^2+^	1.25	
Magnesium	Mg^2+^	0.60	0.75
Sodium	Na^+^	140.00	140.00
Chloride	Cl^−^	115.90	122.00
Hydrogen phosphate	HPO_4_^2−^	1.20	1.00
Hydrogen carbonate	HCO_3_^−^	30.00	22.00
Potassium	K^+^	4.00	4.00
Theoretical osmolarity	mOsm/l	292.95	289.75
pH		7.0–8.5	7.0–8.0

### Data collection

All parameters used for this retrospective study were taken from monitoring parameters collected for clinical routine. Blood gas analysis (BGA) was performed at least four times per day, or as clinically appropriate. Blood sampling was performed once daily. Data about patient’s respiratory situation were routinely collected simultaneously at the BGA sampling time points. Collected data were pseudo-anonymized as soon as possible and further processed in Microsoft Excel (Microsoft Corporation, One Microsoft Way, Redmond, WA 98,052–6399, USA). Study was approved by the Ethics Committee of the Medical University (Approval No. 1070/2018) waiving the requirement of informed consent due to the retrospective nature of the study.

### Statistical analysis

Statistical processing was performed using R (R Core Team 2019, R Foundation for Statistical Computing, Vienna, Austria) and SPSS (IBM SPSS Statistics for Windows, Version 24.0. Armonk, NY: IBM Corp., USA). Baseline demographics, clinical characteristics, and outcomes are presented as the frequency and percentage for categorical and mean ± standard deviation (SD) or median with interquartile range (IQR) for continuous variables. Non-parametric analysis was performed, as appropriate, using the Dunn–Bonferroni post hoc method following a significant Friedman test for repeated measures, for primary and secondary outcomes as well as the sensitivity analysis. The observed effect for the primary endpoints was more precisely examined by Mann–Whitney *U* test including effect size calculation. Since, HCO_3_^−^ and BE were normally distributed (Kolmogorov–Smirnov test) at 0 h and 24 h, differences in means (paired samples *t* test) with 95% confidence interval [CI], effect size measured with Cohen’s *d*_*z*_ and Pearson correlation coefficient was additionally calculated. Effect size interpretation was defined as small (*d* = 0.2, *r* = 0.1), medium (*d* = 0.5, *r* = 0.3) and large (*d* = 0.8, *r* = 0.5) based on benchmarks suggested by Cohen [13].

Between-group comparisons and correlations were analysed between intracorporeal HCO_3_^−^, PaCO_2_ and pH, between minute ventilation and intracorporeal HCO_3_^−^ and PaCO_2_ in mechanically ventilated patients, and in non-mechanically ventilated patients between the respiratory rate and intracorporeal HCO_3_^−^ and PaCO_2_, as appropriate, using Mann–Whitney *U* tests and Spearman correlation coefficients.

The strength of Spearman correlation was defined very high from 0.90 to 1.00, high from 0.70 to 0.90, moderate from 0.50 to 0.70, low from 0.30 to 0.50 and negligible from 0.00 to 0.30 [14]. All tests were 2-sided (significance level 5%).

## Results

**Fig. 1:**
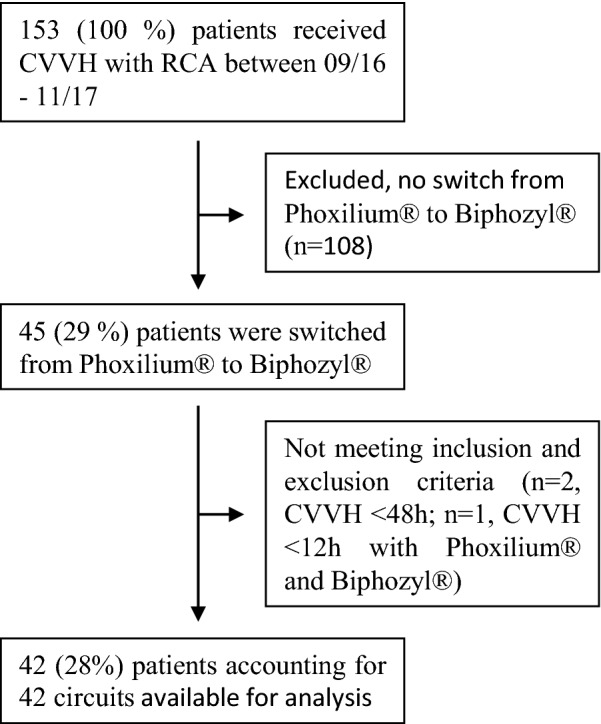
Patients flowchart

A total of 153 patients received CVVH in the ICU during the study period. Of these, 108 (70.6%) patients were not switched from Phoxilium^®^ to Biphozyl^®^ and therefore excluded. The remaining 45 (29.4%) suffered from refractory metabolic alkalosis with a serum HCO_3_^−^ > 26 mmol/l and were switched from Phoxilium^®^ to Biphozyl^®^. After exclusion of 3 further patients because of short treatment periods (*n* = 2, CVVH < 48 h; *n* = 1, CVVH < 12 h with Phoxilium^®^ and Biphozyl^®^), 42 patients accounting for 42 circuits were available for final analysis (see Fig. 1 for patient flow**)**. Of the analysed patients, 15 (35%) were female. The median age was 59.4 (IQR 47.9–69.2) years. Median weight was 74 kg (IQR 65.0–88.5), with a median body mass index of 25.6 (IQR 21.8–30.8). Median CVVH treatment time was 214.7 (IQR 101.2–397.5) hours. Baseline characteristics of the patients are presented in Table 2.

**Table 2:** Baseline characteristics of included patients

Patient characteristics	
Number of eligible patients	42
Age, median (IQR), year	59.4 (47.9–69.2)
Sex, no. (%)	
Male	27 (64)
Female	15 (35)
Weight, median (IQR), kg	74.0 (65.0–88.5)
Body mass index, median (IQR), kg/m_2_	25.6 (21.8–30.8)
Obesity (BMI ≥ 30), no. (%)	11 (27)
Comorbidities, no. (%)	
Diabetes	8 (19)
Chronic obstructive pulmonary disease	4 (9)
Immuno-incompetence	13 (31)
Cardiac failure	8 (19)
Liver failure	15 (35)
End-stage renal disease	3 (7)
Home oxygen/ventilation	1 (2)
Location before ICU admission, no. (%)	
Other hospital ward	19 (45)
Emergency room	15 (35)
Other ICU	8 (19)
Reason for ICU admission, no. (%)	
Cardiovascular/respiratory (e.g. cardiogenic, septic-shock)	35 (83)
Liver failure	7 (16)
Surgical status at ICU admission, no. (%)	
Scheduled surgery	1 (2)
Emergency surgery	5 (11)
No surgery	36 (85)
SAPS 3 at ICU admission, median (IQR)	74.0 58.8–84.0
Duration of CVVH therapy, median (IQR), hours	215 (101–398)
Pre-switch	66 (38–156)
Post-switch	111 (45–335)
Respiratory status during study period, no. (%)	
Mechanical ventilation during whole study period	20 (48)
Mechanical ventilation intermittently during study period	12 (29)
No mechanical ventilation	9 (21)
Study participation, median (IQR), hours	114 (84–144)
ICU stay, median (IQR), days	19 (10–29)
Hospital stay, median (IQR), days	40 (23–63)
Mortality, no./total no. (%)	
ICU	12/42 (28.6)
Hospital	19/42 (45.2)

### Primary outcomes

**Fig. 2:**
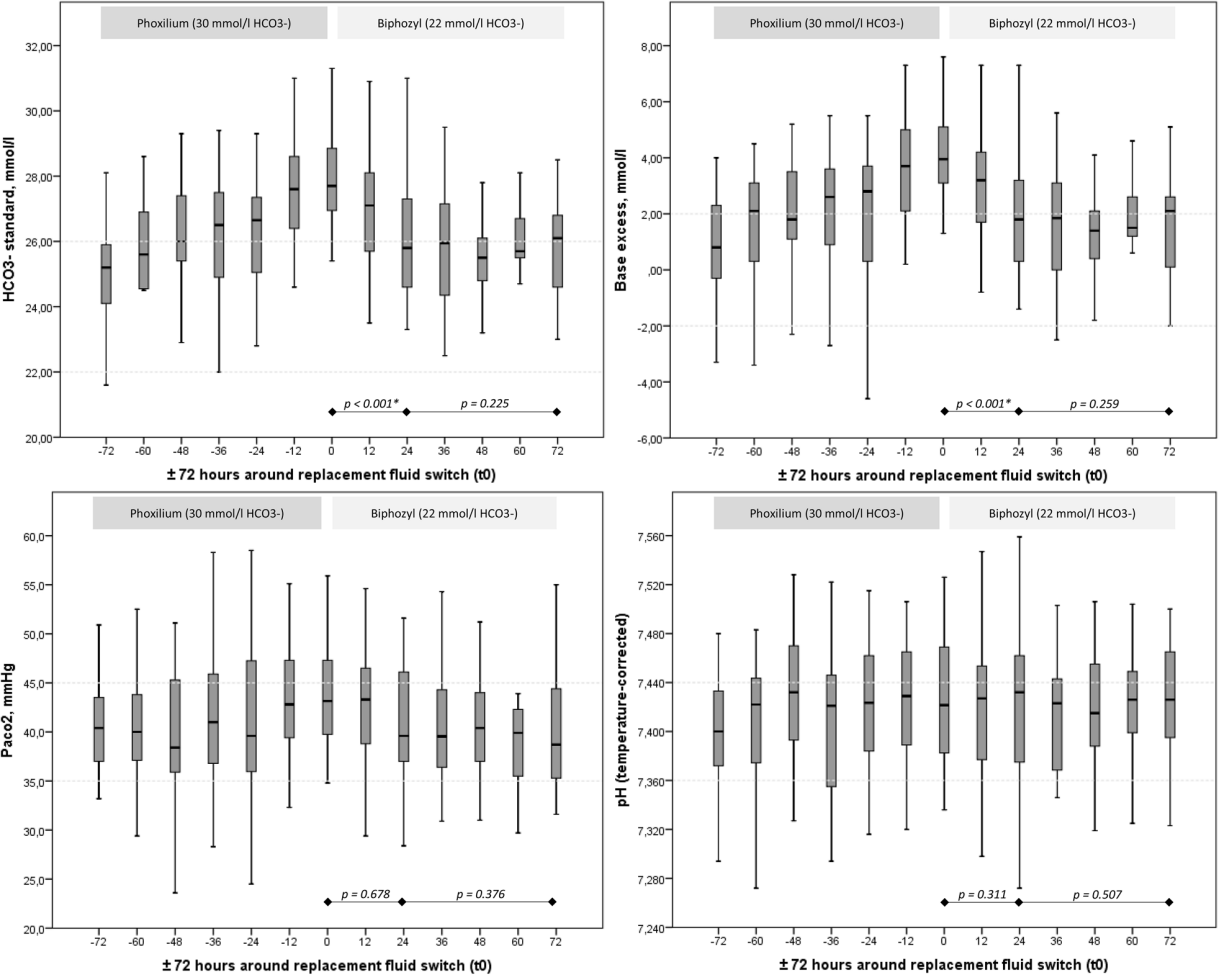
Time course of HCO_3_^−^, BE, PaCO_2_ and pH over 72 h before and after switching (0 h) the replacement fluid from Phoxilium^®^ to Biphozyl^®^

After switching (0 h) the replacement fluid from Phoxilium^®^ to Biphozyl^®^ the HCO_3_^−^ concentration significantly decreased from 27.7 mmol/l (IQR 26.9–28.9) to 25.8 mmol/l (IQR 24.6–27.7) within 24 h (*p* < *0.001*), with a medium to large median difference of 1.8 mmol/l [CI 1.3; 2.4] (effect size, *r* = *−* *0.5*). The BE significantly decreased from 4.0 mmol/l (IQR 3.1–5.1) to 1.8 mmol/l (IQR 0.2–3.4) within 24 h (*p* < *0.001)*, with a medium to large difference of 2.1 [CI 1.5; 2.7] (effect size, *r* = *−* *0.5*). Primary outcomes are presented in Table 3 and Fig. 2. Additional comparison of the means 24 h after the replacement fluid switch further confirmed a significantly large decrease for HCO_3_^−^ (difference 1.813 mmol/l [CI 1.245; 2.380], Cohen’s *d*_*z*_ 1.429, *r* = *0.6*) and BE (difference 2.063 mmol/l [CI 1.453; 2.672], Cohen’s *d*_*z*_ of 1.348, *r* = *0.6*) (see Additional file 1: Fig. S1). The corresponding acid–base parameters, hemofiltration settings as well as the gas exchange and respiratory–ventilation parameters did not change significantly between these time points, see Table 3.

**Table 3:** Acid–base parameters, hemofiltration settings, gas exchange and respiratory/ventilation parameters

Parameter	0 h (at switch)	24 h (after switch)	*p*
	*N* = 42	*N *= 40	
Acid–base parameters			
Bicarbonate, mmol/l	27.7 (26.9–28.9)	25.8 (24.6–27.7)	0.000*
Base excess, mmol/l	4.0 (3.1–5.1)	1.8 (0.2–3.4)	0.000*
pH	7.42 (7.38–7.47)	7.43 (7.37–7.46)	*0.311*
Lactate, mg/dl	12.0 (7.3–14.0)	12.0 (8.0–15.0)	*0.728*
Haemofiltration settings			
Pre-dilution pre-blood pump fluid (Prismocitrate™), ml/h	1200 (1200–1300)	1,273 (1200–1300)	*0.569*
Substitution rate, ml/h	800 (600–1000)	800 (500–1000)	*0.943*
Fluid removal, ml/h	100 (100–188)	100 (100–150)	*0.543*
Blood flow, ml/min	120 (125–130)	120 (120–130)	*0.884*
Filtration fraction, %	33 (30–36)	33 (30–35)	*0.799*
Temperature, °C	41.0 (40.0–43.0)	41.0 (39.8–43.0)	*0.863*
Citrate dose, mmol/l	3.0 (3.0–3.2)	3.0 (3.0–3.2)	*0.127*
Ionized serum calcium, mmol/l	1.09 (1.03–1.15)	1.06 (1.00–1.12)	*0.170*
Calcium dose, mmol/h	2.45 (2.10–2.88)	2.65 (2.28–3.20)	*0.196*
Calcium substitution, %	90 (85–105)	100 (88–111)	0.000*
Post-filter calcium, mmol/l	0.41 (0.37–0.43)	0.37 (0.36–0.40)	0.006*
Gas exchange and respiratory/ventilation parameters			
PaCO_2_, mmHg	43.2 (39.7–47.5)	39.6 (36.8–46.1)	*0.678*
PaO_2_, mmHg	82.9 (70.1–95.7)	84.2 (71.4–97.2)	*0.691*
Peripheral arterial oxygen saturation, %	97.0 (93.7–98.3)	96.8 (95.5–98.3)	*0.767*
PaO_2_/FiO_2_ ratio, mmHg	227 (131–298)	213 (136–328)	*0.834*
Ventilator parameters	*N* = 25	*N* = 23	
Respiratory rate, breaths/min	17 (14–20)	17 (15–20)	*0.971*
FiO_2_	0.35 (0.30–0.50)	0.35 (0.30–0.53)	*0.861*
Tidal volume, mL/kg PBW^a^	6.3 (5.8–6.9)	6.5 (5.9–7.8)	*0.511*
Set positive end-expiratory pressure, cmH2O	8 (8–10)	8 (8–10)	*0.733*
Peak pressure, cmH2O	19 (16–21)	18 (16–21)	*0.529*
Driving pressure, cmH2O	10 (7–12)	9 (8–12)	*0.510*
Minute ventilation, mL/kg PBW^a^/min	105 (85–123)	111 (85–135)	*0.511*
Non-invasive ventilation parameters	*N* = 17	*N* = 17	
FiO_2_	0.50 (0.23–0.68)	0.40 (0.21–0.49)	*0.177*
Respiratory rate, breaths/min	16 (15–18)	20 (13–24)	*0.404*

Data reported as median (IQR), unless otherwise specified

^a^PBW predicted body weight

### Secondary outcomes

HCO_3_^−^ (*p* = *0.225*) and BE (*p* = *0.259*) concentrations remained stable from 24 h after the replacement fluid change till the end of observation (72 h). For PaCO_2_ a decreasing trend could be observed from 43.2 mm Hg (IQR 39.7–47.5) to 39.6 mm Hg (IQR 36.8–46.1) within the first 24 h after the replacement fluid switch (*p* = *0.678)*. The pH did not change significantly (*p* = *0.311)* from 7.42 (IQR 7.38–7.47) to 7.43 (IQR 7.37–7.46) during that time. See Table 3 and Fig. 2 for results. The sensitivity analysis of 26 patients who completed treatment from − 24 h to + 72 h confirmed the significant reduction of HCO_3_^−^ (*p* = *0.025*) and BE (*p* = *0.011*) within 24 h after the replacement fluid switch, which remained constant during the following 48 h (presented in Additional file 2: Fig. S2).

Correlation analysis over the whole study period showed, that the intracorporeal HCO_3_^−^ correlated significantly with PaCO_2_ (*p* = *0.001*, coefficient = *0.181)* and pH (*p* < *0.001*, coefficient = *0.456).* PaCO_2_ and pH correlated significantly (*p* < *0.001, *coefficient = *0.723*) as well. Minute ventilation correlated significantly with intracorporeal HCO_3_^−^ (*p* = *0.002, *coefficient = *−* *0.221)* and PaCO_2_ (*p* = *0.019, *coefficient = *−* *0.167)* in mechanically ventilated patients but at negligible strength*.* In non-mechanically ventilated patients, the respiratory rate significantly correlated with intracorporeal HCO_3_^−^ (*p* = *0.022, *coefficient = *−* *0.239)* and PaCO_2_ (*p* = *0.006, *coefficient = *−* *0.301)* at a low to negligible level. Although a significant difference of the median pH was apparent between mechanically ventilated and non-invasive ventilated patients at the switch of the replacement fluid (0 h) (7.42 vs. 7.47, *p* = *0.02*) and 24 h thereafter (7.41 vs. 7.46, *p* = *0.002*), no significant change occurred in either group over 72 h after switching the replacement fluids.

It is important to mention that Biphozyl^®^ does not contain Ca^2+^. Therefore, we evaluated the calcium homeostasis after the switch of the replacement fluid as a safety endpoint. For ionized serum Ca^2+^ (1.09 mmol/l vs. 1.06 mmol/l, *p* = *0.170*) and the Ca^2+^ dose (2.45 mmol/h vs. 2.65 mmol/h, *p* = *0.196*), no significant change could be observed between 0 and 24 h. The Ca^2+^ substitution significantly increased (90.0% vs. 100.0%, *p* < *0.001)* after 24 h. From 24 h until 72 h after the replacement fluid switch ionized serum Ca^2+^ (1.06 mmol/l vs. 1.08 mmol/l, *p* = *0.057*) did not change significantly. Ca^2+^ dose (2.65 mmol/h vs. 2.80 mmol/h, *p* = *0.023*) and Ca^2+^ substitution (100.0% vs. 105.0%, *p* < *0.033*) significantly increased during this time.

## Discussion

This investigation demonstrates that the use of a replacement fluid with low bicarbonate content normalizes refractory metabolic alkalosis and achieves sustained bicarbonate levels within normal range in critically ill patients treated with CVVH and RCA.

Increased bicarbonate levels during CVVH using RCA is a frequent finding [8, 15], which potentially increases the risk of mortality [12]. In our cohort of patients, 29.4% showed metabolic alkalosis defined by blood HCO_3_^−^ concentrations ≥ 26 mmol/l. Lower rates were reported by Jeffrey et al. who found that during CVVH with RCA 8.3% of the patients developed alkalosis defined by pH ≥ 7.5 [11] which, however, is not only dependent on HCO_3_^−^ but strongly influenced by pCO_2_ levels in the patients.

Switching the replacement fluid from Phoxilium^®^ (30 mmol/l HCO_3_^−^) to Biphozyl^®^ (22 mmol/l HCO_3_^−^) resulted in significantly lower HCO_3_^−^ and BE concentrations within 24 h followed by constant levels during the next 48 h. This suggests that a ‘new’ steady state was reached 24 h after exchanging replacement fluids. Apparently Biphozyl^®^, with its lower bicarbonate levels, leads to a reduction of metabolic alkalosis in patients treated with CVVH and RCA.

To date, few studies have compared Phoxilium^®^ with other replacement fluids. While sample size of most trials was small, they could show an effect on pH and bicarbonate levels, when comparing Phoxilium^®^ to replacement fluids with higher bicarbonate concentrations (Hemosol^®^ B0, 32 mmol/l; multiBic^®^, 35 mmol/l; AccusolTM, 35 mmol/l) [16–20]. All trials, that compared Phoxilium^®^ with replacement fluids containing higher bicarbonate levels, reported significantly lower serum bicarbonate levels during CRRT treatment with Phoxilium^®^. The results for pH were heterogeneous. For pCO_2_, none of the studies reported a significant decrease. Most trials either used no anticoagulation or heparin or prostacyclin/epoprostenol [16–18, 20], only one study used RCA [11].

Most existing RCA protocols recommend that either the adjustment of the blood flow or the replacement fluid/dialysate rate should be applied to correct acid–base disturbances during CRRT [21–25]. More specifically, a decrease of blood flow and/or an increase of replacement/dialysate fluid flow are considered as sufficient options to correct alkalosis, especially, if the most common modalities CVVHDF (continuous veno-venous hemodiafiltration) or CVVHD (continuous veno-venous hemodialysis) are applied. However, the latter option is not effective, if a high HOC_3_^−^ containing substitution fluid is used. Thus, we aimed at evaluating an alternative approach for treatment of metabolic alkalosis when using purely convective CVVH with RCA. Based on our findings, we consider switching the replacement fluid from high- to low-bicarbonate-containing solutions as an appropriate method which does not alter treatment dose. We want to emphasize, however, that our findings are only applicable for CVVH and probably not relevant for CVVHDF, if low bicarbonate-containing solutions are used as dialysate.

Whether there is a clinically relevant direct correlation between the amount of administered HCO_3_^−^ and pCO_2_ blood levels, can only be speculated. In our patients, HCO_3_^−^ and PaCO_2_ correlated at negligible strength*.* The pH seemed to be determined more by PaCO_2_ than by HCO_3_^−^ in our cohort*.* Former experimental and clinical studies showed an increase in pCO_2_ with a parallel rise in pH after the administration of bicarbonate in specific situations [26, 27]. However, this could not be demonstrated for a cohort of ventilated patients undergoing CVVH recently [28]. Also, in patients with acute respiratory distress syndrome (ARDS) no differences in arterial CO_2_ tension or in tidal or minute ventilation were observed for different severities of AKI including requirement of RRT [29]. Thus, it remains unclear whether reducing HCO_3_ load by CVVH will have a relevant impact on CO_2_ generation in critically ill patients.

Finally, we found some dynamics in ionized serum Ca^2+^ levels within the following 72 h after the change of replacement solutions due to the fact that Biphozyl^®^ does not contain Ca^2+^. Acute hypocalcaemia might occur and lead to neuromuscular irritability or even cardiac manifestation. Thus, we would like to emphasize the importance of close monitoring of patients’ ionized serum Ca^2+^ levels for adequate substitution. Due to our experience and awareness of the lower concentration of calcium within Biphozyl^®^, we increased Ca2 + substitution rates accordingly and did not observe any significant problems.

### Limitations

Our study is prone to the typical weaknesses associated with a retrospective trial (e.g. selection bias, information bias, inability to investigate parameters other than those previously collected during clinical routine, etc.). Secondly, we could not control for differences in several pre-hospital and hospital factors: (a) evidence of previous kidney function/dysfunction (except RRT-dependent AKI); (b) any RRT within the previous 2 months; (c) main criterion for CVVH indication, and (d) presence of adverse events. Furthermore, our patients differed in severeness of organ failure (single/multi) and primarily affected organ system (cardiovascular/respiratory and liver failure patients). Third, in our sample only 9 (21%) patients were not mechanically ventilated, which limits generalizability for this subgroup. Fourth, as not all patients received CVVH from − 72 h to + 72 h, not all patients contributed data over the whole observation period. However, a sensitivity analysis using a sample of 26 patients who completed 72 h confirmed our findings. Finally, our study may lack external validity by not being applicable to other modalities like CVVHDF or patients who do not develop metabolic alkalosis during RCA.

### Strengths

Our study has several strengths. First, this is the first trial that evaluated Biphozyl^®^ and the second trial that evaluated Phoxilium^®^ during CRRT with RCA. It is not only among the largest studies on this subject, but also covers the longest observation period, comprising 72 h before and after the switch of replacement fluid. Secondly, no significant changes of the blood flow and replacement fluid flow rate occurred during the observed decline in HCO_3_^−^ and BE. This is of great importance, as these are the only substantial confounders for the effect established by the switch of the replacement fluid. Hence, our results may be considered robust within this clearly defined cohort.

## Conclusions

This retrospective analysis suggests that for patients receiving CVVH with RCA who develop metabolic alkalosis refractory to adjustments of CVVH settings, the use of Biphozyl^®^ reduces external HCO_3_^−^ load and sustainably corrects intracorporeal HCO_3_^−^ and BE concentrations. Future studies have to prove whether this replacement solution is suitable for maintaining normal acid–base status in all critically ill patients requiring CVVH with RCA. Our current prospective, randomized, controlled, open, cross-over, Phase II study (BiPhox-Trial; ClinicalTrials.gov Identifier: NCT04071171) should provide answers to this question. Ultimately, it remains to be proven whether correcting metabolic alkalosis during CVVH is of clinical relevance with regard to patient outcome.

## Supplementary Information

**Additional file 1: Figure S1.** Changes of HCO_3_^−^ and BE between the switch of the replacement fluid and 24 h thereafter. Mean Difference (Δ), Confidence Interval (CI) 95%, effect size measure measured with Cohen’s d_z_ and Pearson correlation coefficient.

**Additional file 2: Figure S2.** Sensitivity analysis comprising 26 patients with a complete dataset from −24 h to +72 h.

## Data Availability

The datasets generated and analysed during the current study are available from the corresponding author on reasonable request.
