# Supplementary material for: Low bicarbonate replacement fluid normalizes metabolic alkalosis during continuous veno-venous hemofiltration with regional citrate anticoagulation
Source: Ann Intensive Care. 2021 Apr 23;11:62. doi: 10.1186/s13613-021-00850-4 (PMC8062940; doi:10.1186/s13613-021-00850-4)
Supplement: Supplementary file 1 — Additional file 1: Figure S1. Changes of HCO3− and BE between the switch of the replacement fluid and 24 h thereafter. Mean Difference (Δ), Confidence Interval (CI) 95%, effect size measure measured with Cohen’s dz and Pearson correlation coefficient. [file 13613_2021_850_MOESM1_ESM.pdf]

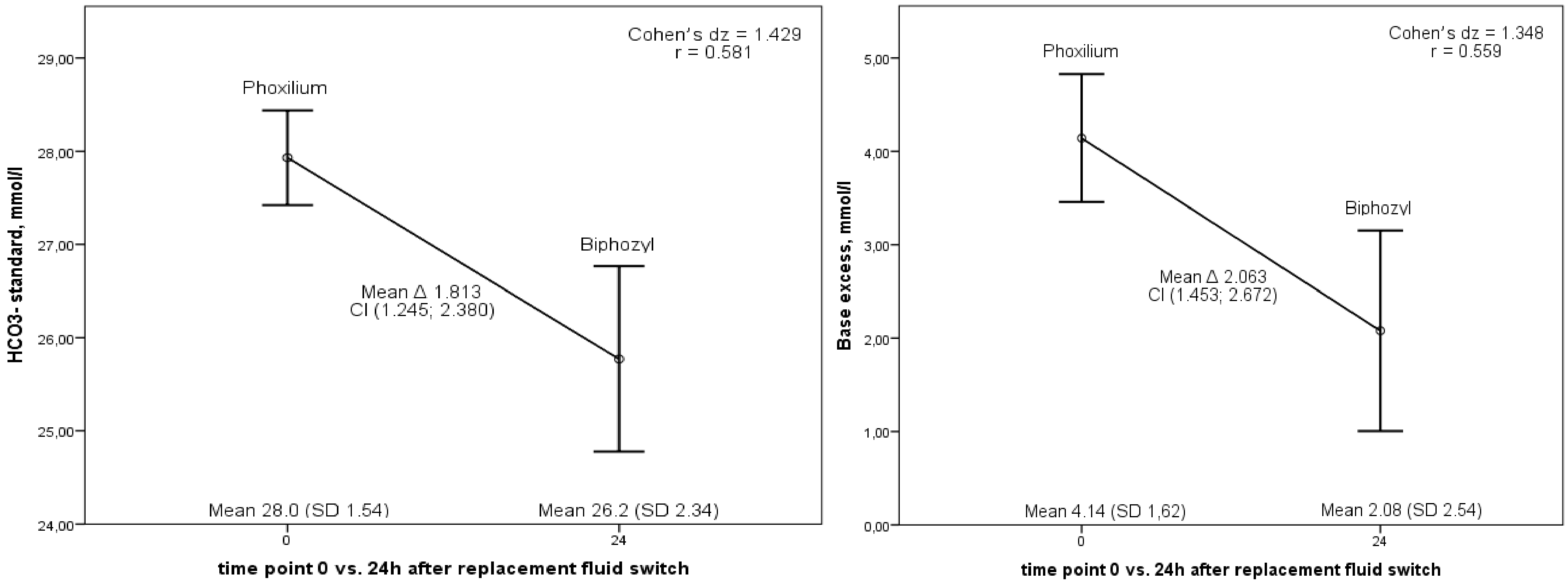

Figure S1. Change of HCO_3^- and BE between the switch of the replacement fluid and 24h thereafter. Mean Difference (Δ), Confidence Interval (CI) 95%, effect size measure measured with Cohen's d_z and Pearson correlation coefficient.
