# Supplementary figures and images for: Low bicarbonate replacement fluid normalizes metabolic alkalosis during continuous veno-venous hemofiltration with regional citrate anticoagulation
Source: Ann Intensive Care. 2021 Apr 23;11:62. doi: 10.1186/s13613-021-00850-4 (PMC8062940; doi:10.1186/s13613-021-00850-4)

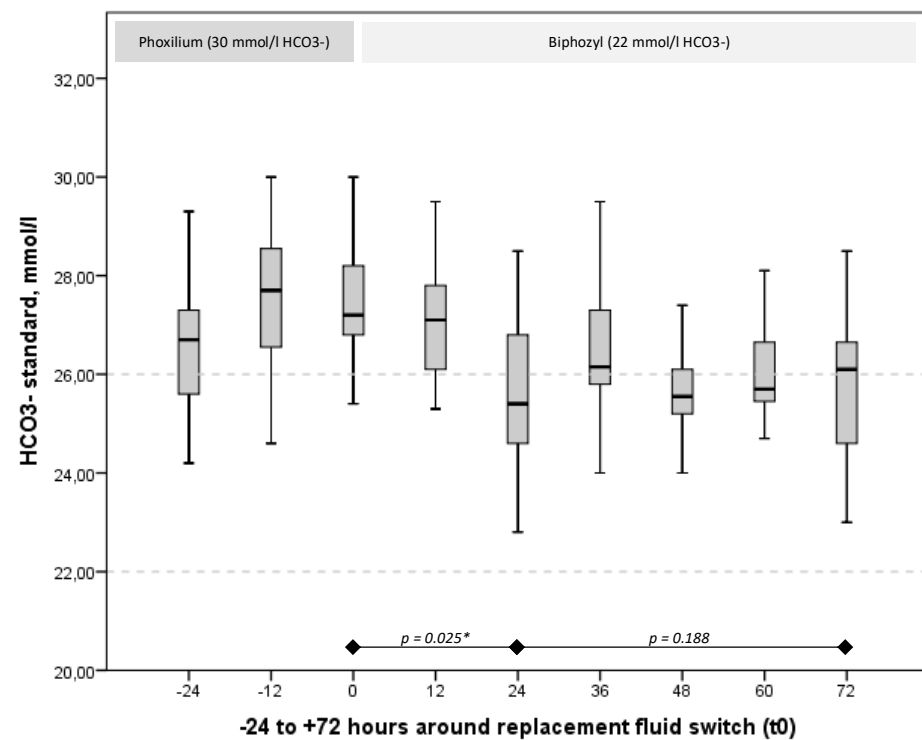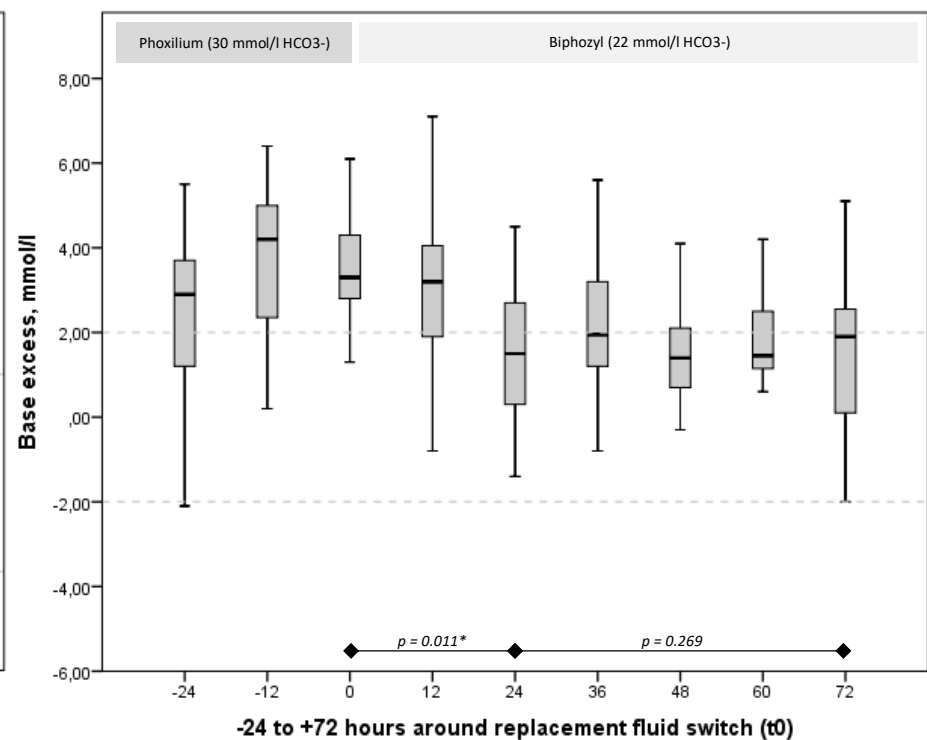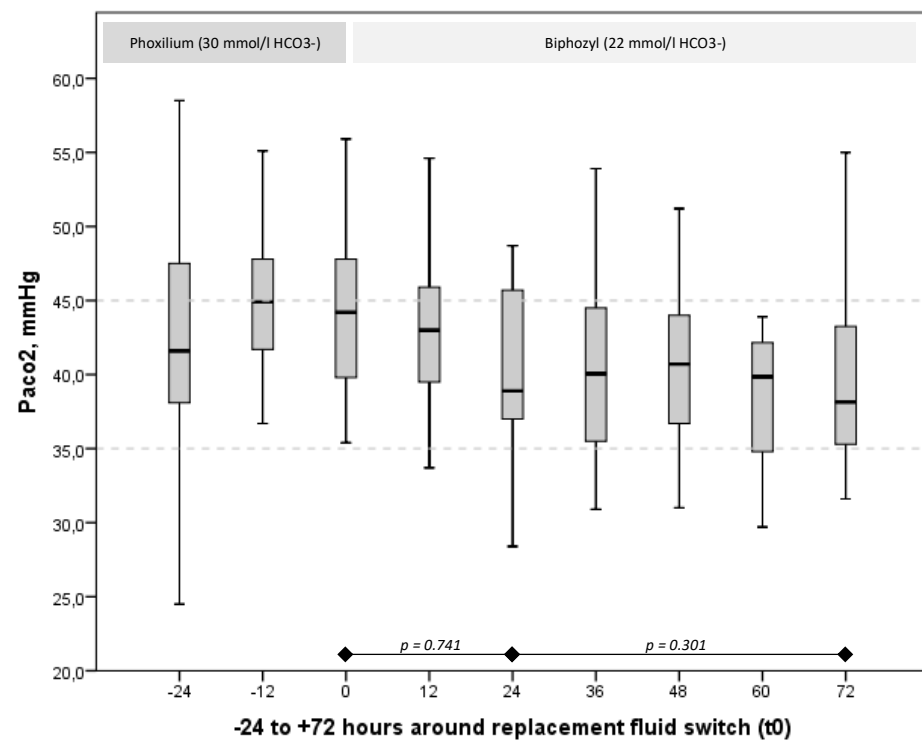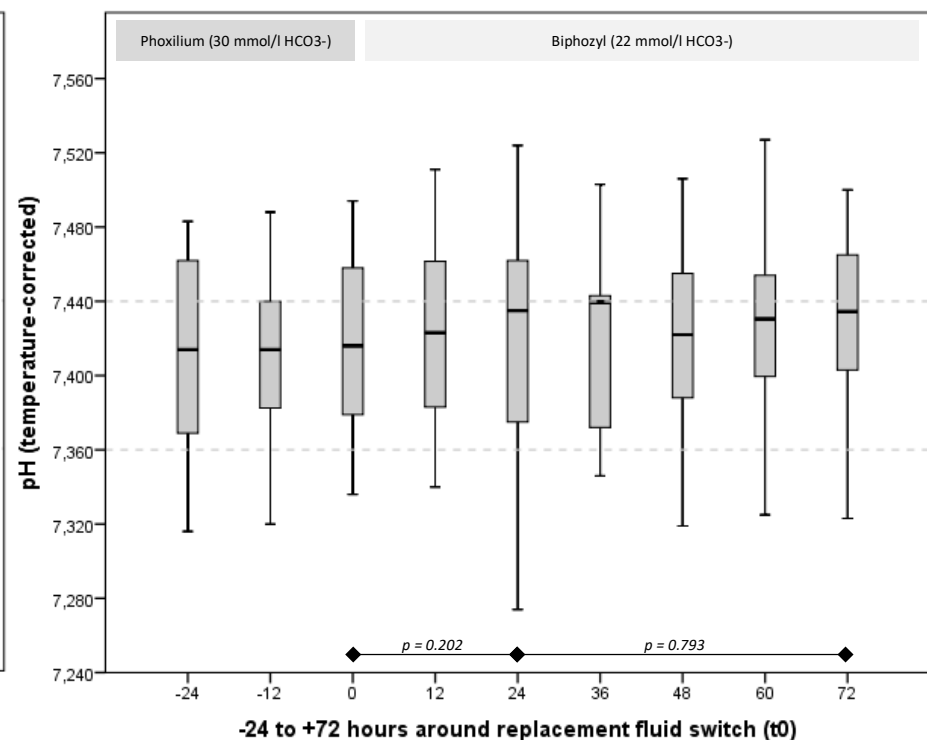

Figure S2. Sensitivity analysis comprising 26 patients with a complete dataset from -24h to +72h.

Supplement: Supplementary file 2 — Additional file 2: Figure S2. Sensitivity analysis comprising 26 patients with a complete dataset from −24 h to +72 h. [file 13613_2021_850_MOESM2_ESM.pdf]
